# Mechanical, Thermal and X-Ray Shielding Properties of Lead-Free Composites of HDPE Filled with Metal-Based Powders

**DOI:** 10.3390/polym18070893

**Published:** 2026-04-06

**Authors:** Sitah Alanazi, Shahad Alshadokhi, Eid Alosime, Mansour Almurayshid, Mohammed Alsuhybani, Mohammad Marashdeh

**Affiliations:** 1 Department of Physics, College of Sciences, Imam Mohammad Ibn Saud Islamic University (IMSIU), P.O. Box 90950, Riyadh 11623, Saudi Arabia; 2 King Abdulaziz City for Science and Technology (KACST), P.O. Box 6086, Riyadh 11442, Saudi Arabia

**Keywords:** lead-free material, polymers, metal-based fillers, X-ray shielding, radiation attenuation

## Abstract

The increasing utilization of radiation in medicine, industry, and water purification highlights the need for efficient radiation-protection materials. This study investigates lead-free polymer composites based on high-density polyethylene (HDPE) filled with four metallic fillers: tungsten carbide (WC), molybdenum carbide (MoC), tungsten (W), and molybdenum (Mo) at 15 wt%. The objective is to evaluate their potential as alternatives to lead for shielding ionizing radiation. Mechanical performance was examined through tensile testing, while thermal stability was assessed based on the residual mass. Radiation-attenuation behavior was analyzed through linear and mass attenuation coefficients (µ and µₘ), radiation protection efficiency (RPE), half-value layer (HVL), mean free path (MFP), buildup factors (B), and effective atomic number (Z_eff_) within the 47.9–248 keV energy range. The HDPE/W composite exhibited the greatest enhancement, with a mass attenuation coefficient (µₘ) 82.5% higher than that of pure HDPE, along with the highest linear attenuation coefficient (µ). Furthermore, tungsten-loaded samples achieved an RPE of 98.05% at 47.9 keV. The increased density, low B, and high Z_eff_ values collectively contribute to superior shielding performance. These findings indicate that HDPE filled with WC, MoC, W, and Mo are promising lead-free candidates for low-energy X-ray shielding applications.

## 1. Introduction

Radiation refers to a form of energy that can travel through space and penetrate substances. Radiation, depending on the ionization of matter, is categorized as ionizing or non-ionizing [1]. It is an important tool in many sectors, such as aerospace, water decontamination and medical applications, including oncology and diagnostic imaging [2,3]. Although radiation provides benefits to patients and workers in various applications, it also poses adverse health effects. Health hazards due to radiation include radiation sickness and increased incidence of cancer [2,4,5]. As explained by the World Health Organization (WHO), even low-level radiation exposure can increase health risks. It is always better to take protective measures from radiation [6].

Three fundamental principles underpin radiation protection: justification, optimization, and limitation of doses [7]. Shielding is a technique to reduce the intensity of radiation with suitable materials. Lead has been used traditionally because of its high density and atomic number, which efficiently attenuates the X-ray and gamma-ray energy [4,7]. Lead has several disadvantages. First of all, it is toxic. Second, lead is heavy. In addition, lead will not shield neutron radiation but will generate secondary ionizing radiation [2,5,8]. The limitations of these materials have inspired the search for safer, lighter-weight, and more versatile alternatives.

Effective shielding against both gamma and neutron radiation typically requires combining high-atomic-number (high-Z) elements, which attenuate gamma rays, with low-atomic-number (low-Z) materials that efficiently scatter and absorb neutrons [8,9,10]. In practical multilayer shielding designs, the low-Z layer is typically placed on the inner side to slow down and thermalize neutrons, whereas the high-Z layer is positioned externally to effectively attenuate gamma rays and minimize secondary gamma generation. A composite material can be defined as a combination of two or more distinct materials, in which the resulting structure exhibits properties superior to those of its individual constituents [11]. High-density polyethylene (HDPE), a polymer, is an attractive material due to its light weight, chemical stability, ease of processing, and excellent neutron-moderating properties [6,12,13,14]. Most shielding materials for ionizing radiation are materials characterized by high electron and mass density.

HDPE is an inexpensive material. Use of high-Z fillers like tungsten (W), tungsten carbide (WC), molybdenum (Mo), and molybdenum carbide (MoC) can improve the effectiveness of the HDPE-based shields and plates. However, it should be noted that elemental W and Mo can undergo significant neutron activation; in this study they are mentioned only as general high-Z fillers and not as recommended materials for neutron-rich environments. When designing successful composite shields, it is important to take into account the filler material, the type and energy range of the radiation, and the device’s use.

The aim of this study is to evaluate lead-free, polymer-based composites as efficient, lightweight, and environmentally benign materials for radiation shielding. These composites are designed to overcome the intrinsic drawbacks of conventional lead shields, such as toxicity, brittleness, and excessive weight, while maintaining comparable or superior attenuation performance. By integrating high-Z fillers into polymer matrices, it is possible to achieve a balanced synergy of mechanical flexibility, processability, and radiation attenuation. Accordingly, we investigate high-density polyethylene (HDPE) composites filled with W, WC, Mo, and MoC at a fixed loading of 15 wt%, selected as an optimal level within the commonly effective 10–20 wt% range to promote uniform dispersion, ensure mechanical stability, and avoid agglomeration [15,16]. Using a single, consistent filler fraction enables a fair and direct comparison of the shielding capabilities of these four reinforcements within an identical matrix. The composites’ resistance to mechanical and thermal stresses and their shielding effectiveness are assessed through low-energy X-ray attenuation coefficients, radiation protection efficiency, buildup factors, and effective atomic numbers.

## 2. Materials and Methods

### 2.1. Materials

Tungsten (W) from H.C. Starck, Goslar, Germany and molybdenum (Mo) from American Elements, Los Angeles, CA, USA, were selected as fillers due to their excellent radiation shielding properties. Tungsten, with an atomic number (Z) of 74 and a density of 19.3 g/cm^3^, achieves effective attenuation at lower thicknesses compared to lead, exhibiting a smaller half-value layer. Being non-toxic further enhances its appeal. Nevertheless, its high cost limits widespread application.

Molybdenum, with a density of 10.2 g/cm^3^ and Z of 42, exhibits minimal toxicity to humans, although it is toxic to certain animals [17]. Their carbide forms (WC, Kennametal Inc., Latrobe, PA, USA and MoC, Goodfellow Cambridge Ltd, Huntingdon, Cambridgeshire, UK) were also considered to further explore enhancements in mechanical and shielding performance. HDPE was used as the polymer matrix. HDPE with a filler loading of 15 wt% was selected, as prior research [2] demonstrated that this concentration provides the best shielding efficiency. A constant filler loading of 15 wt% was used in all HDPE-based composites to ensure comparability and isolate the influence of filler type (W and Mo) on X-ray attenuation properties. This concentration was selected as an effective compromise between attaining enough attenuation efficiency and preserving acceptable processability and mechanical integrity. Prior research indicates that high filler concentrations (exceeding around 20 wt%) often lead to particle agglomeration, inadequate interfacial bonding, and a decline in mechanical performance [18]. Kaya et al. [18] similarly identified an ideal filler threshold, beyond which the composite’s structural and mechanical qualities deteriorate owing to non-uniform dispersion and void formation. Moreover, higher filler loadings significantly enhance melt viscosity and decrease the melt flow index, complicating processing and achieving uniform mixing in HDPE systems with metallic fillers [19]. Consequently, a 15 wt% filler concentration was deemed an optimal mid-range loading to reconcile radiation shielding efficacy with manufacturing practicality. Future research should explore various filler concentrations (e.g., 5, 10, and 20 wt%) to determine the best ratio for certain shielding applications.

### 2.2. Sample Preparation

HDPE was obtained from SABIC (Saudi Basic Industries Corporation, Riyadh, Saudi Arabia). WC, MoC, W, and Mo were incorporated at a concentration of 15 wt% within the HDPE matrix. The formulations and corresponding sample codes are listed in Table 1: H-1 (pure HDPE), HWC-2 (HDPE + WC), HMoC-3 (HDPE + MoC), HW-4 (HDPE + W), and HMo-5 (HDPE + Mo).

HDPE was preheated in the mixing chamber of a Brabender Plasticorder (Duisberg, Germany) for 4 min until fully molten. The fillers were then gradually introduced while maintaining a temperature of 180 °C and a screw speed of 60 revolutions per minute (rpm). Mixing proceeded for 10 min to ensure complete homogenization.

The blend was subsequently transferred to a preheated two-roll mill (Brabender) and processed at 170 °C with a roll speed of 20 rpm for another 10 min to form uniform sheets. These sheets were hot-pressed using a Collin P 400 p.m. press (COLLIN Lab & Pilot Solutions GmbH, Maitenbeth, Germany) at 170 °C and 150 bar for 10 min, with the full pressure applied only during the final 2 min of the cycle.

Finally, the composite sheets were cut into circular specimens with a diameter of 20 mm and a thickness of 2 mm using a CEAST cutting machine (Instron, Pianezza, Italy).

### 2.3. Density Measurement

The density of the samples was determined using the Archimedes principle with ethanol as the immersion medium. Measurements were performed using an analytical balance (Mettler Toledo GmbH, Greifensee, Switzerland). Each sample’s mass was recorded both in air ($m_{a}$) and in ethanol ($m_{l}$), and the density (ρ sample) was calculated using the following formula:

(1) $$\rho =\frac{m_{a}}{m_{a}-m_{l}}\left(\rho_{l}-\rho_{air}\right)+\rho_{air}$$

where $\rho_{l}$ is the density of the auxiliary liquid, $\rho_{air}$ is the density of air, $m_{a}$ is the mass of the sample in air, and $m_{l}$ is the mass of the sample in the auxiliary liquid [20].

### 2.4. Tensile Testing

The tensile properties of the samples were evaluated using a universal testing machine (Instron 5982, Grove City, PA, USA) in accordance with the ASTM D638 standard [21]. Dumbbell-shaped specimens with a thickness of 2 mm were prepared from the composite sheets.

The testing was conducted at a crosshead speed of 50 mm/min, with an applied force ranging between 32 and 365 N. Each sample was stretched until fracture, and the tensile strength, Young’s modulus, and elongation at break were recorded. Data acquisition and analysis were performed using Bluehill 3 software (version 3.15.1343).

Five measurements were conducted for each composite type, and the results were averaged to ensure reliability. All mechanical properties reported are presented as mean ± standard deviation (SD) based on five replicates (n = 5).

### 2.5. Analysis of Thermal Stability

The thermal stability of the composites was evaluated using a thermogravimetric analyzer (TGA 1, PerkinElmer, Shelton, CT, USA). The samples were heated from room temperature to 800 °C at a constant heating rate of 10 °C/min under a nitrogen atmosphere. TGA and DTG, or derivative thermogravimetry, were performed to examine the thermal degradation behavior and to estimate the filler loading based on the high-temperature residual mass. The weight-loss curves were recorded as a function of temperature, and the residual mass at 700–800 °C was compared with the theoretical filler content to verify consistency.

### 2.6. Evaluations of X-Ray Shielding Parameters

This study evaluates attenuation performance under narrow-beam X-ray conditions using linear and mass attenuation coefficients (μ, µₘ). Dose-based or biological shielding endpoints are not considered; therefore, results are not reported in Gy.

The evaluation of the shielding performance of the composites was done by determining the linear attenuation coefficient (µ), the mass attenuation coefficient (µₘ), the mean free path (MFP), the half-value layer (HVL), the tenth-value layer (TVL), the radiation protection efficiency (RPE), the buildup factor (B), and the effective atomic number (Z_eff_).

The μ, in cm^−1^, describes the probability of photon interactions per unit path length in the material and is given by:

(2) $$\mu =\frac{1}{x}ln(\frac{Io}{I})$$

where Io and I are the incident and transmitted photon intensities, respectively, and x is the sample thickness (cm) [22,23].

The µₘ (cm^2^/g) is obtained by normalizing µ by the sample density (ρ) [24]:

(3) $$\mu_{m}=\;\frac{\mu }{\rho }$$

The MFP is an important variable in determining the µ. Simply put, it tells what average distance a photon is able to travel in the absorber before an interaction occurs [23,25].

(4) $$\mu =\frac{1}{MFP}$$

The HVL is the thickness of the absorbing substance that diminishes the beam intensity to half of its initial value [22,26]. The correlation between the half-value layer x1/2 and the attenuation coefficient μ can be established by:

(5) $$HVL=x_{1/2}=\frac{ln2}{\mu }$$

The TVL is the thickness of the absorbing material that attenuates the beam intensity to one-tenth (10%) of its original intensity as following:

(6) $$TVL=x_{1/10}=\;\frac{ln10}{\mu }$$

Furthermore, RPE can be utilized to assess the shielding efficacy of composite samples according to the µ, as denoted by the subsequent relation;

(7) $$\;RPE(\%)=\left(1-\frac{I}{Io}\right)\times 100$$

The B in radiation measurement is the ratio of the total radiation quantity, such as photon fluence, photon energy fluence, exposure, or dose, after traversing a medium, to the radiation amount that reaches at the point without interaction [25,27]. The B can be expressed as:

(8) $$\left.\;B=\frac{I}{Io{(e}^{\mu x})}\right.$$

where *I* is the number of photons counted by detector, and Io the total number of photons emitted from source.

Z_eff_ correlates with an element’s density and atomic number and therefore cannot denote a singular atomic number throughout the entire energy spectrum. For specific applications, the Z_eff_ is employed to indicate the atomic number of a compound that lacks uniqueness. Z_eff_ is utilized to define interactions with photons, and it is correlated with (µ/ρ) and can be articulated by the following equation:

(9) $$Z_{eff}=\;\left.\frac{\Sigma i\;ni\;Ai\;(µ/\rho )i}{\Sigma i\;ni\;(Ai\;/Zi)\;(µ/\rho )i}\right.$$

where n_i_ is a number of atoms present in the ith element present in a molecule, Z_i_ is the atomic number of the ith element present in a molecule, A_i_ is the atomic mass of the ith element, and (µ/ρ)_i_ is the mass attenuation coefficient of ith element present in the molecule [28].

### 2.7. Experiment Setup

**Figure 1:**
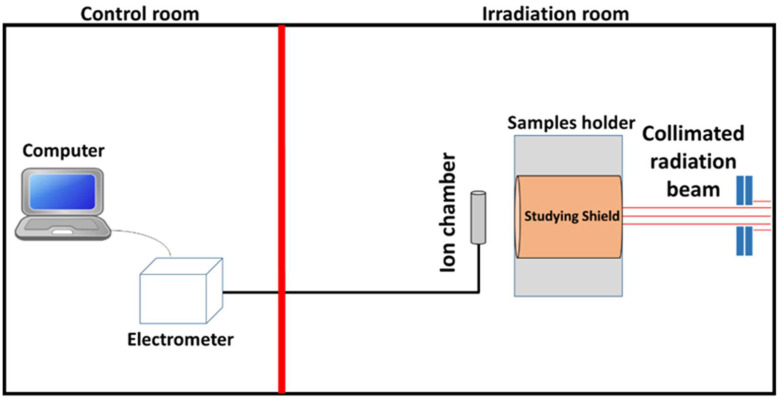
Experimental setup at the radiation calibration laboratory (KACST), showing the control room and the irradiation room.

The X-ray shielding performance of five different HDPE-based composites was evaluated at the radiation calibration laboratory, King Abdulaziz City for Science and Technology (KACST), Riyadh, Saudi Arabia. The laboratory includes a control room and an irradiation room separated by a lead-shielded door, as shown in Figure 1.

Samples were mounted in a dedicated holder positioned in front of the X-ray tube. The X-ray tube used in this study employed a tungsten anode, which is the standard target material for producing broad-spectrum diagnostic X-rays at high tube potentials. The X-ray system was equipped with a filter wheel to select the desired beam quality, and the beam intensity was controlled remotely from the control room. Radiation intensities were measured using a PTW 23361 ionization chamber (PTW, Freiburg, Germany) connected to a PAM electrometer (Budapest, Hungary), with data acquisition performed using Pico Ampere Meter W2006 (PAMW2006) software.

The X-ray beam setup was tightly controlled to ensure reproducibility, following the ISO 4037-1 narrow-beam geometry [29]. A fixed source-to-sample distance of 100 cm was used, and the beam was collimated to a 10 × 10 cm field at the sample position. All filters followed the standard ISO 4037-1 configurations and were selected through the system’s filter wheel. The beam was aligned horizontally toward the ionization chamber, and all measurements were performed under identical geometric conditions.

Measurements were performed at tube potentials of 60, 80, 100, 120, 150, 200, 250, and 300 kV, as detailed in Table 2. In accordance with ISO 4037-1 procedures, the incident photon-energy distribution was defined by the tube voltage together with the specified filtration, and the resulting beam quality was verified using the measured HVL. The effective energies in Table 2 are the ISO 4037-1 values for each N-series beam, representing the mean photon energy defined by the tube voltage, filtration, and verified HVL.

For each composite material, 15 disk samples with a diameter of 20 mm and a thickness of 2 mm were stacked and exposed to the X-ray beam. The 15 disks were placed in a dedicated alignment holder and clamped under a fixed, repeatable setting to minimize interlayer air gaps and ensure a constant total thickness. The stack thickness was verified before each run, and the same assembly and fixing conditions were used for all measurements. The X-ray beam was directed horizontally through the center of the sample stack toward the ionization chamber. The axial gap between the sample and the chamber was 10 cm, with a positioning repeatability of ±1 mm. The chamber reference point was the geometric center of the collecting volume, as specified by the manufacturer.

**Figure 2:**
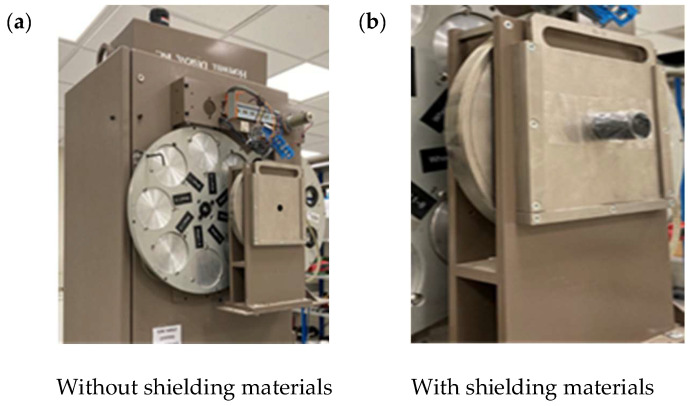
Measurement setup for evaluating X-ray attenuation: (**a**) incident beam measurement without the sample (I_0_), and (**b**) transmitted beam measurement with stacked HDPE composite disks in the beam path.

The incident beam intensity (I_0_) was first measured without any sample present. Subsequently, the transmitted intensity (I) was recorded with the samples in place. Three measurements were conducted at each energy level, and the average value was used for analysis. Each measurement was taken for 10 s to ensure stable readings and consistent statistical uncertainty. The µ and µₘ were calculated based on the measured intensities. The experimental setup is illustrated in Figure 2.

## 3. Results and Discussion

### 3.1. Density Measurement

The density of the HDPE composites was measured experimentally and compared with theoretical predictions based on the filler content. All composites contained a constant 15 wt% of WC, MoC, W, or Mo, as detailed in Table 3.

The incorporation of fillers led to an increase in composite density compared to pure HDPE. The experimental density values ranged from 0.957 g/cm^3^ (pure HDPE) to 1.102 g/cm^3^ (HDPE + 15% WC). The composite containing WC exhibited the highest density, with a 15.15% increase over pure HDPE. The HDPE composite with 15% W showed a density of 1.091 g/cm^3^, corresponding to a 14% increase.

The comparison between experimental and theoretical densities revealed a strong agreement. In Table 3, “Error (%)” represents the percentage difference between the experimental and calculated density values for each composite. The maximum deviation between experimental and theoretical values was 1.49% for the HDPE + W composite, while the minimum deviation was 0.35% for HDPE + WC. These small error ratios confirm the homogeneity of filler dispersion and the reliability of the preparation method.

Overall, the density measurements validate the successful incorporation of fillers into the HDPE matrix, resulting in composites with enhanced density suitable for radiation shielding applications. These density values agree with previous studies on HDPE composites filled with high-Z fillers such as Bi_2_O_3_ and Gd_2_O_3_, where a 10–20% increase in density improved shielding performance [16,30]. This consistency supports the reliability of the preparation method and demonstrates alignment with established polymer-based shielding studies.

### 3.2. Tensile Testing

Table 4 presents the tensile strength, elongation at break, and Young’s modulus of the HDPE composites containing 15 wt% of WC, MoC, W, and Mo particles. The tensile strength and elongation at break of the composites generally decreased compared to pure HDPE, which indicates a reduction in both ductility and tensile properties. This behavior is attributed to limited interfacial bonding between the rigid filler particles and the HDPE matrix, leading to stress concentration points and more brittle fracture mechanisms. Similar reductions in ductility along with increases in stiffness have also been reported in metal-particle-reinforced polyolefins [31]. In these systems, rigid particles act as stress concentrators and limit chain mobility, as demonstrated in HW-4 composites. Our results follow the same trend, with the magnitude of the effect varying depending on filler dispersion quality.

Conversely, an increase in Young’s modulus was observed for the HW-4 and HMo-5 composites, with values of 2000 MPa and 2150 MPa, respectively. The enhancement in stiffness is primarily due to the high rigidity and uniform dispersion of W and Mo particles, which effectively restrict polymer chain mobility and reinforce the composite matrix [25]. Representative stress–strain curves are provided in the Supplementary Materials (Figure S1).

**Figure 3:**
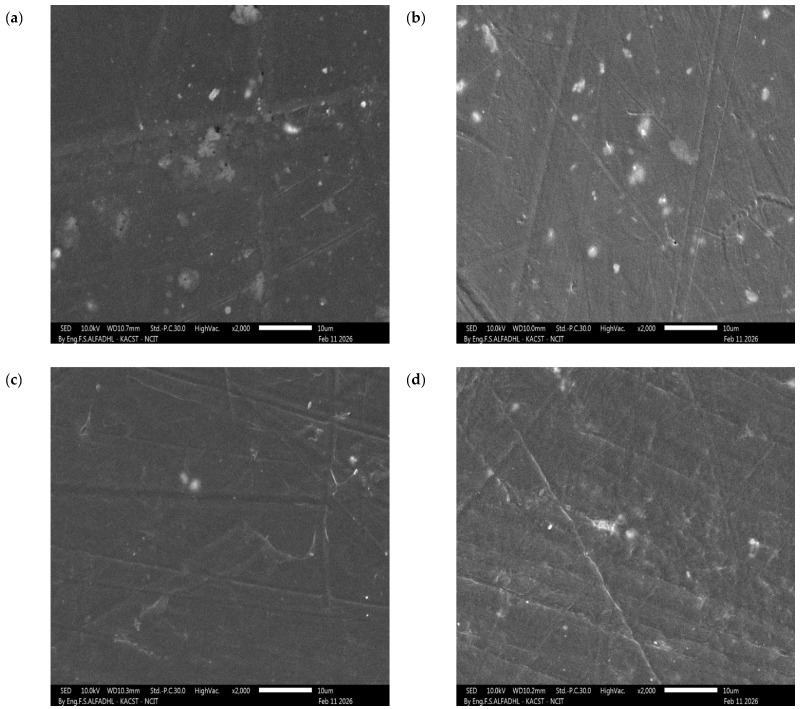
SEM micrographs of HDPE composite samples: (**a**) HWC-2, (**b**) HMoC-3, (**c**) HW-4, and (**d**) HMo-5.

Interestingly, the HW-4 composite (HDPE + tungsten) also exhibited a relatively higher elongation at break compared to other filled composites. The lower tensile strength of HW-4 is likely related to stress concentration and limited interfacial adhesion introduced by rigid W particles. However, the higher elongation at break suggests a possible toughening effect when the filler is well dispersed, which can delay crack propagation and enhance energy absorption prior to failure [1,32]. This suggests that the well-dispersed W particles may contribute to improved energy absorption and toughness, allowing the material to undergo greater deformation before fracture. This is supported by the observed uniform dispersion in SEM (Figure 3).

Overall, the results indicate that the incorporation of metal-based particles into HDPE significantly enhances stiffness while reducing ductility, which is a characteristic behavior of particle-reinforced polymer composites.

### 3.3. Morphology

The surface morphology of the HDPE composites was examined using SEM. Figure 3 presents representative micrographs of HWC-2, HMoC-3, HW-4, and HMo-5, while additional details regarding the local W and Mo atomic density obtained from SEM-EDS are provided in the Supplementary Materials (Figures S2–S6). Composites containing 15 wt% Mo and MoC (Figure 3b,d) exhibit a more uniform dispersion of particles within the HDPE matrix, with minimal signs of clustering. In contrast, the addition of 15 wt% W and particularly WC (Figure 3a,c) leads to regions of higher contrast and noticeable textural variability, indicating minor particle agglomeration and less homogeneous packing within the observed field. Such differences in dispersion quality may partly explain the variations observed in density and mechanical behavior among the composites. It is also possible that partial particle settling occurred during melt processing due to differences in density, chemical structure, and interfacial compatibility between HDPE and the metal-based fillers; these factors can reduce the effectiveness of mixing and promote local clustering, especially for high-density fillers such as W and WC. Additionally, limited interfacial bonding during the heating–cooling cycle may contribute to the formation of larger agglomerates in the 15 wt% composites, which could negatively affect overall performance, underscoring the importance of minimizing agglomeration to achieve improved properties [30].

### 3.4. Thermo-Gravimetric Analysis (TGA)

**Figure 4:**
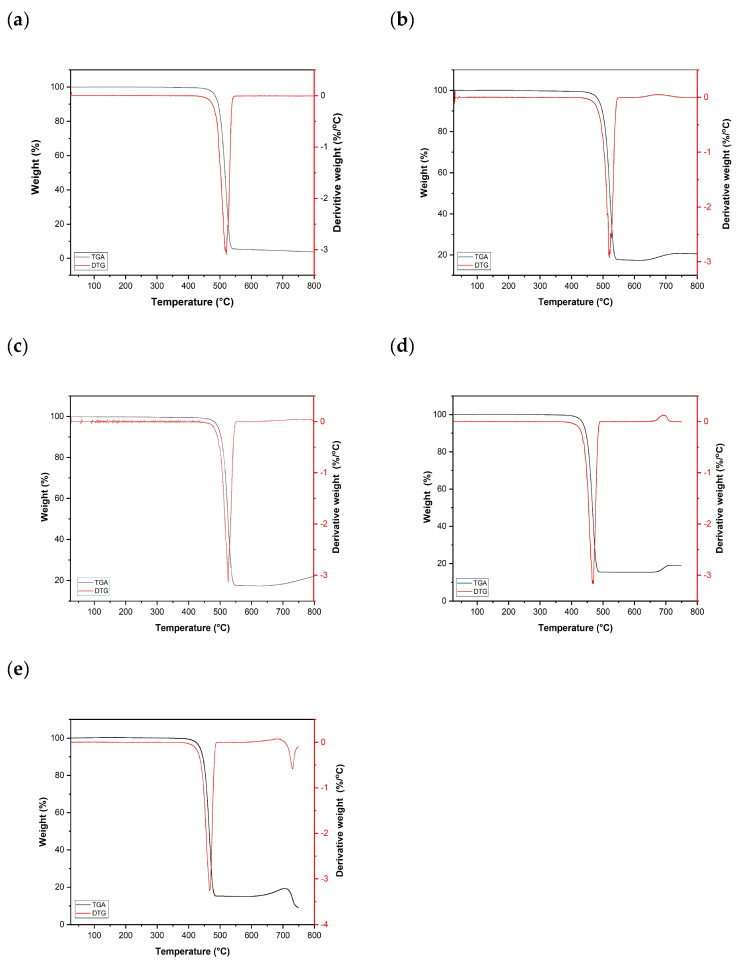
TGA/DTG analysis curves of (**a**) H-1 (**b**) HWC-2, (**c**) HMoC-3, (**d**) HW-4, and (**e**) HMo-5, showing weight-loss behavior as a function of temperature.

The thermal stability of HDPE composites was investigated using TGA over a temperature range from 25 °C to 800 °C. Figure 4 displays the TGA curves of pure HDPE and HDPE composites containing up to 15 wt% of various fillers.

Pure HDPE exhibited high thermal stability, with negligible weight loss observed up to an onset degradation temperature of approximately 503.03 °C. The onset temperature marks the beginning of significant polymer decomposition. HDPE mainly decomposes by random C–C chain scission with subsequent β-scission to volatile hydrocarbons; the metal-based fillers do not decompose in this range and remain as residue [32].

As shown in Figure 4 and summarized in Table 5, the composites demonstrated residual weights ranging from 18% to 20% at 700 °C. In contrast, the non-zero residual mass observed for pure HDPE (4.44%), despite the theoretical value of 0%, is not a true solid residue but arises from trace impurities and inherent TGA measurement limitations during random-scission thermal degradation. The slight apparent weight increases above ~700 °C is attributed to high-temperature TGA artifacts (e.g., buoyancy and baseline drift), rather than a real mass gain; therefore, reporting residue at 600 °C is more reliable [16,33]. This residual mass corresponds to the presence of thermally stable inorganic fillers (W, WC, Mo, and MoC) remaining after the decomposition of the polymer matrix. The consistency of the residual weight with the nominal filler content (15 wt%) confirms the effective incorporation and distribution of the fillers within the HDPE matrix. These results are consistent with the density measurements and theoretical predictions, further validating the composite preparation process.

### 3.5. Shielding Parameters Analysis

**Figure 5:**
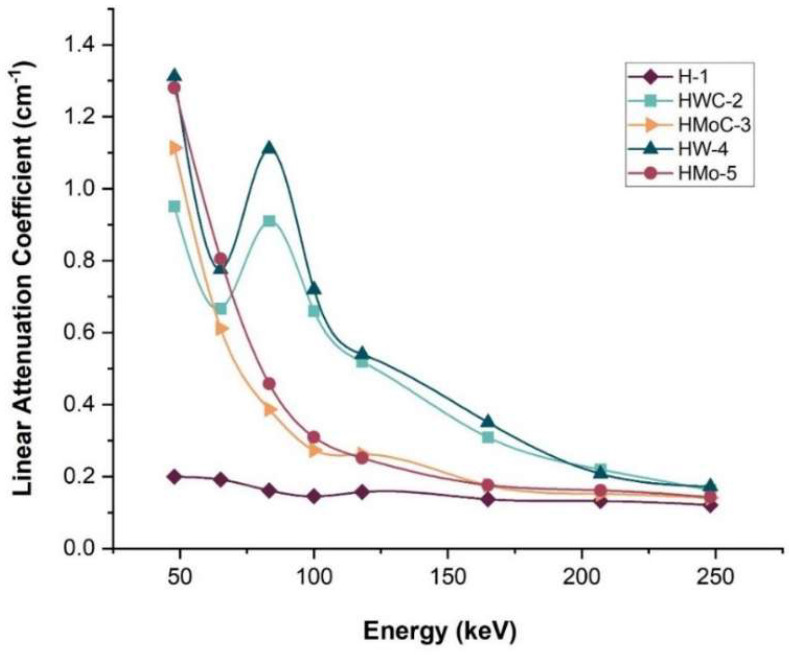
Measured linear attenuation coefficient (µ) as a function of X-ray photon energy for HDPE polymer composites.

The X-ray attenuation performance of the HDPE composites was evaluated by measuring the µ under narrow-beam conditions across tube voltages ranging from 60 to 300 kV. Figure 5 presents the variation in µ with photon energy for pure HDPE and the composites containing 15 wt% fillers.

The results indicate that pure HDPE exhibited the lowest attenuation coefficients across the entire energy range, while the HW-4 composite (HDPE + 15% W) demonstrated the highest µ values. The incorporation of high-atomic-number fillers significantly enhanced the radiation shielding efficiency of the HDPE matrix compared to the unfilled polymer. The observed trend agrees with previous studies indicating that photon attenuation in polymer composites is mainly governed by the atomic number and density of the fillers. Unlike earlier research focused on Bi- or Pb-based fillers, this study systematically compares four metallic fillers under identical conditions, offering clearer insight into filler-dependent shielding behavior [34]. The small peak observed around 80–100 keV in the attenuation curves is attributed to the transition between dominant photon-interaction mechanisms. At low energies, attenuation is mainly governed by the photoelectric effect, whereas at medium energies Compton scattering becomes more significant. This shift in dominant interactions produces a local maximum in the curves and is consistent with the expected behavior of Mo- and W- filled HDPE composites.

**Figure 6:**
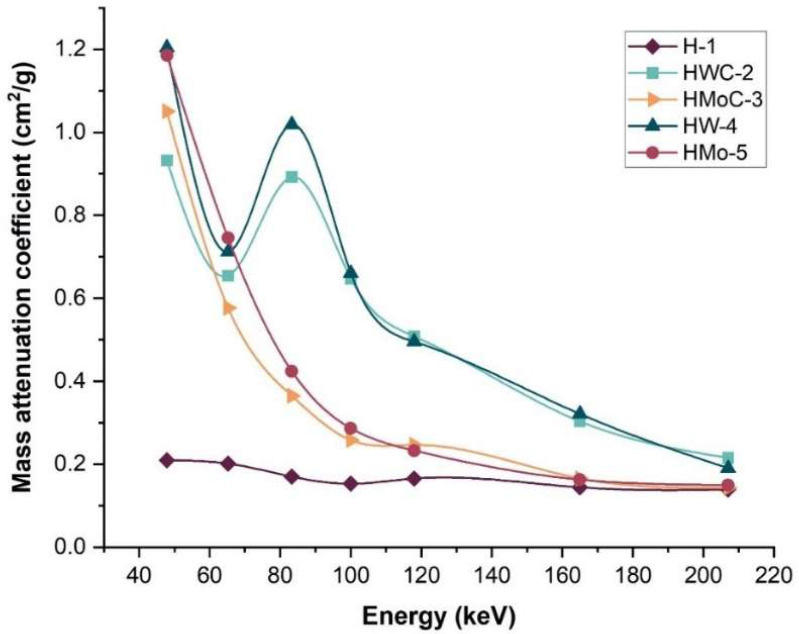
Measured mass attenuation coefficient (µₘ) as a function of X-ray photon energy for HDPE polymer composites.

To further assess shielding performance, the µₘwere experimentally determined and compared with theoretical values obtained from the XCOM database (NIST). As shown in Table 6 and illustrated in Figure 6, the HW-4 composite exhibited the highest µₘ, exceeding that of pure HDPE by approximately 82.5%. The experimental µₘ for HW-4 deviated from the theoretical XCOM value by only 2.2%, indicating good agreement and confirming the reliability of the experimental methodology. It is also worth noting that SRIM was not employed in this study because it simulates only charged particles and does not model photon-interaction mechanisms such as the photoelectric effect or Compton scattering. For X-ray and gamma-ray energies, the XCOM database (NIST/ORNL) is the standard and most appropriate tool for theoretical photon attenuation coefficients; therefore, the comparison between the experimental µ/ρ values and XCOM provides an accurate and reliable validation of the shielding performance of the prepared composites.

The Mo–HDPE and W–HDPE composites have different attenuation properties due to variations in atomic number and density. Tungsten, with a higher atomic number (Z = 74) and density (19.3 g/cm^3^), consistently exhibits superior attenuation compared to molybdenum (Z = 42; 10.2 g/cm^3^) at all evaluated photon energies. This confirms tungsten’s great efficacy as a high-Z filler for enhancing photon interactions inside the polymer matrix. Regarding shielding efficacy, both fillers enhance the attenuation of HDPE: tungsten is notably successful at increased attenuation efficiency throughout a wide energy spectrum, whilst molybdenum provides moderate shielding that may be beneficial in applications requiring reduced material weight. Collectively, our findings illustrate that selecting between Mo and W fillers enables customization of the composite for certain shielding applications, optimizing performance, weight, and cost factors.

Although the filler content in all composites is limited to 15 wt%, the observed enhancement in X-ray attenuation is scientifically consistent with the underlying interaction mechanisms. Photon attenuation, particularly at low and medium energies, is dominated by the photoelectric effect, whose probability scales approximately with Z^3^/E^3^, making the contribution of high-Z fillers disproportionately strong compared to their volume fraction. Thus, fillers such as W (Z = 74) and Mo (Z = 42) markedly increase µ and µ/ρ even when added in relatively small weight percentages.

In contrast, density, tensile strength, and elongation at break scale primarily with the filler volume fraction and the efficiency of load transfer at the filler–matrix interface, which change only modestly at 15 wt%. Mechanical properties are therefore not expected to exhibit the same magnitude of change as radiological parameters. This inherent difference between atomic-number-driven radiation interactions and volume-fraction-driven mechanical behavior explains why the attenuation coefficients increase significantly while the mechanical and physical properties vary only slightly. This distinction aligns with findings reported in high-Z polymer-matrix shielding systems and supports the validity of the experimental interpretation. The observed deviation for HWC-2 at 83.3 keV is primarily attributed to the transition between photoelectric and Compton interactions, which enhances sensitivity to small physical variations, in addition to experimental uncertainties.

### 3.6. Radiation Protection Efficiency (RPE)

The RPE of the HDPE composites was evaluated based on the measured µ over a photon energy range of 47 to 248 keV. The RPE values for all samples are summarized in Table 7. According to the analysis, RPE generally decreases with increasing photon energy, which was also concluded in other studies [35]. This decrease occurs because photoelectric absorption, which dominates at lower energies, becomes less effective at higher energies. The property of photoelectric absorption at lower energies is fairly strong, resulting in enhanced radiation attenuation, which results in a high RPE value. Among the composite materials tested, the HW-4 sample (which consisted of HDPE combined with 15% W) was found to have the greatest RPE value, achieving a value of 98.05% at an energy level of 47.9 keV. This impressive performance indicates the good quality of tungsten-based composites in absorbing and attenuating low-energy X-rays. The high RPE of HW-4 demonstrates that even a moderate loading of tungsten can significantly enhance the shielding efficiency of polymer composites. Since diagnostic radiology procedures typically use photon energies below 100 keV, the HW-4 composite appears to have the potential to be used in medical X-ray shielding applications. However, this conclusion is limited to transmission-based attenuation in the studied energy range, and further evaluation under clinically relevant scatter conditions, repeated exposure, and different dose rates is required before confirming medical applicability. On the other hand, pure HDPE has the least radiation protection performance, with an RPE of 45.00% at 47.9 keV and further dropping to 30.47% at 248 keV, highlighting the importance of high-Z fillers in improving attenuation. These results clearly show that the choice and concentration of fillers strongly influence the RPE and that low-energy X-rays are more effectively blocked by materials containing dense elements like tungsten. The enhanced shielding performance is attributed to: (i) the high atomic number of the fillers, which increases photoelectric absorption—particularly at low photon energies where attenuation scales approximately with Z^3^/E^3^; (ii) the higher composite density, which raises the interaction probability per unit path length; and (iii) particle dispersion, as uniform distribution improves the effective interaction cross-section while agglomeration reduces shielding efficiency. This explains the superior performance of W-filled HDPE (Z = 74; 19.3 g/cm^3^) compared with Mo-based fillers.

### 3.7. Half-Value Layer (HVL)

In further assessment of the attenuation efficiency of the HDPE composites, the HVL was also calculated, which is the material thickness that would eliminate half of the incident radiation beam. The µ values were used to determine the HVL values through Equation (6).

**Figure 7:**
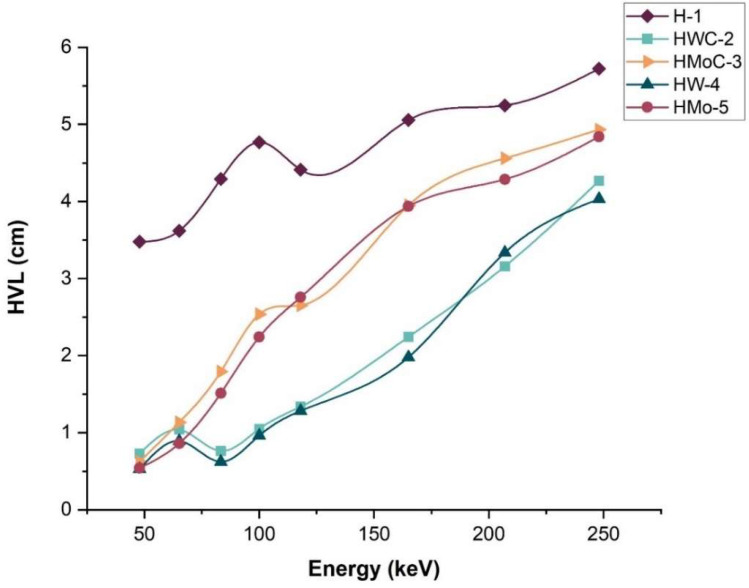
Variation in HVL values for HDPE polymer composites as a function of photon energy.

Figure 7 displays the HVL results of pure HDPE and the composites within a range of photon energy from 47.9 to 248 keV. As predicted, the HVL increased with the advancement of photon energy since the penetration capacity of high-energy photons is higher.

HWC-2 and HW-4 (HDPE + 15% W) had the lowest HVL among the composites. The HVL of HW-4 (HDPE + 15% W) was found to be 0.53 cm at 47.9 keV, 0.96 cm at 100 keV, and 4.03 cm at 248 keV. These results indicate that a smaller material thickness is required to achieve 50% attenuation when using the HW-4 composite compared to other composites and pure HDPE, confirming its superior shielding performance.

### 3.8. Mean Free Path (MFP)

The MFP represents the average distance a photon travels within a material before undergoing an interaction. MFP is inversely proportional to the µ, as described by Equation (5). A lower MFP indicates a higher probability of photon interaction, reflecting greater shielding effectiveness.

**Figure 8:**
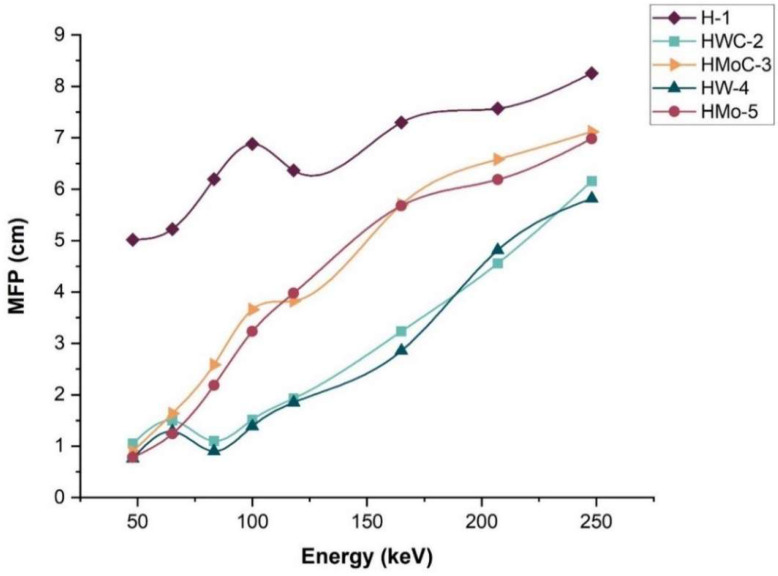
Mean free path (MFP) values for pure HDPE and HDPE composites containing 15 wt% of W, WC, Mo, and MoC fillers.

Figure 8 presents the MFP values for pure HDPE and the composites as a function of photon energy. The findings reveal that pure HDPE has the highest values of MFP across all energies, indicating less efficient attenuation. The explanation is because both tungsten-based composites, HW-4 (HDPE + W) and HWC-2 (HDPE + WC), have the lowest MFP values amongst the samples, meaning photon interaction with the medium is more likely in these two, leading to better shield performance as indicated by their higher linear attenuations.

These results show that adding high-atomic-number fillers to HDPE increases the chance of photon interaction, which may improve shielding effectiveness.

### 3.9. Buildup Factors (B)

The B is an important parameter that is commonly used in the design and evaluation of radiation shielding materials. It takes into account the contribution of scattered and secondary photons to the overall dose. Radiation protection engineers and physicists may face difficulties from having high B, which may cause dose leakage through shielding.

**Figure 9:**
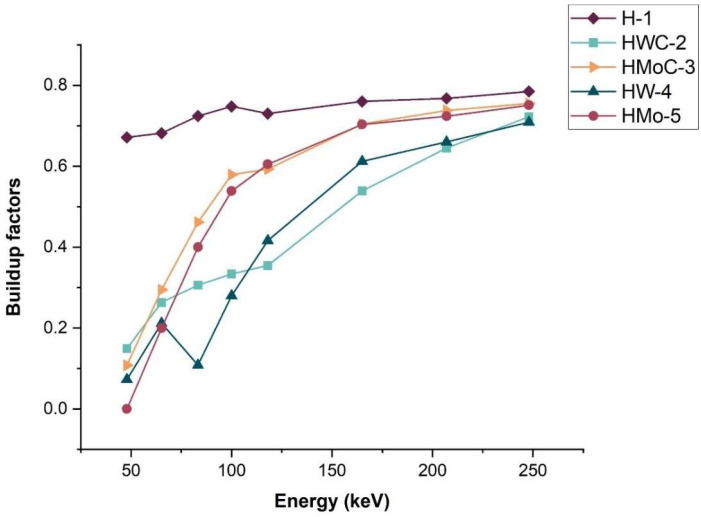
Measured buildup factors (B) for HDPE polymer composites.

The Bs for pure HDPE and composites are shown in Table 8 and Figure 9. An increase in photon energy leads to an increase in B, which infers that with an increase in energy, the probability of scattering also increases.

Among all samples, HWC-2 and HW-4 composites exhibited the lowest B across the energy range studied (47.9–248 keV), with a value of approximately 0.71 at 248 keV for HW-4. This indicates a lower contribution of scattered radiation and a higher shielding efficiency compared to other composites and pure HDPE.

These findings are consistent with previous studies [36,37], which reported that materials with lower B, such as polyvinylidene chloride (PVDC), exhibit superior shielding performance. The superior performance of HW-4 can be attributed to the high atomic number and density of tungsten, enhancing both photon absorption and scattering suppression.

### 3.10. Effective Atomic Number (Z_eff_)

The Z_eff_ is a key parameter for characterizing the interaction of photons with composite materials. Unlike pure elements, composites cannot be represented by a single atomic number across the entire energy range. Instead, an effective atomic number is calculated based on the fractional contributions of each constituent element [38,39,40].

**Figure 10:**
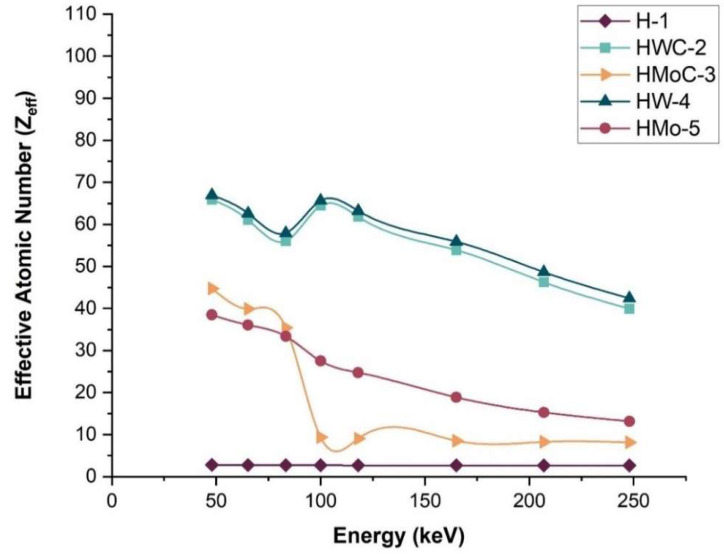
Variation in the calculated effective atomic number (Z_eff_) with incident photon energy for the samples within the selected energy range.

The scattering and absorption of X-rays are closely related to both the material’s density and its Z_eff_. As shown in Table 9 and illustrated in Figure 10, the Z_eff_ of the composites varies with photon energy, reflecting the different interaction mechanisms at different energies. The sharp decrease in Z_eff_ for HMoC-3 at higher photon energies is attributed to the transition from photoelectric-dominant interactions at low energies to Compton-dominant interactions at medium energies. Since Compton scattering is far less sensitive to atomic number, Z_eff_ values for MoC-filled composites drop rapidly and converge toward lower values as energy increases.

Among the composites, HW-4 (HDPE + 15% W) exhibited the highest effective atomic number across the studied energy range. Z_eff_ is the main driver of the improved shielding, particularly at low photon energies where attenuation strongly depends on atomic number, while density plays a secondary supporting role. This indicates superior shielding capability, particularly at lower photon energies where the photoelectric effect dominates, with interaction probability approximately proportional to Z^4.5^ and inversely proportional to E^3^.

These results confirm that the incorporation of high-atomic-number fillers, such as tungsten, significantly enhances the X-ray attenuation performance of HDPE-based composites. Unlike previous studies focusing on a single filler, this work systematically compares four metallic fillers (W, WC, Mo, MoC) at identical loadings and processing conditions, evaluated using calibrated narrow-beam X-ray standards (ISO 4037). This unified framework enables direct comparison of structure–property–shielding relationships, providing new insight into how filler type, dispersion, and density collectively influence attenuation performance.

## 4. Conclusions

This study investigated whether incorporating 15 wt% of metallic fillers (W, WC, MoC, Mo) into HDPE under identical processing and calibrated ISO 4037-1 narrow-beam X-ray conditions can produce a lightweight, lead-free composite with enhanced low-energy X-ray shielding while maintaining acceptable thermal and mechanical performance.

Across 47.9–248 keV, W-filled HDPE consistently exhibited the highest μ and μ/ρ, the lowest HVL and MFP, and the highest RPE and Z_eff_, with experimental μ/ρ values closely matching XCOM predictions (≤2.5% deviation). These findings follow the expected attenuation mechanism, where low-energy photon absorption strongly depends on atomic number and composite density, while uniform dispersion enhances the effective interaction cross-section and agglomeration reduces efficiency.

Filler incorporation increased density (up to 1.102 g·cm^−3^ for 15% WC) and preserved thermal stability (~503 °C, single-stage decomposition). As typical for particle-reinforced polyolefins, stiffness increased whereas tensile strength and ductility decreased, with variations governed by dispersion quality observed in SEM/EDS.

Overall, HDPE + 15 wt% W provides the most favorable balance among the tested systems for low-energy X-ray shielding. Unlike prior studies evaluating a single high-Z filler, this work offers a unified comparative framework linking structure, dispersion, density, and attenuation performance. Future studies should extend evaluation to broad-beam geometries, optimize thickness and filler content, and improve interfacial adhesion to enhance mechanical resilience without compromising shielding efficiency.

## Figures and Tables

**Table 1:** Composition and sample codes of HDPE composites with different fillers (wt%).

Formulation Code	Composition (wt%)
H-1	Pure HDPE
HWC-2	HDPE (85%) + WC (15%)
HMoC-3	HDPE (85%) + MoC (15%)
HW-4	HDPE (85%) + W (15%)
HMo-5	HDPE (85%) + Mo (15%)

**Table 2:** X-ray narrow-beam qualities used in the experiment, including tube potentials and their corresponding effective photon energies according to ISO 4037-1.

Shortened Name	Tube Potential (kV)	Effective Energy (keV)
N-60	60	47.9
N-80	80	65.2
N-100	100	83.3
N-120	120	100
N-150	150	118
N-200	200	165
N-250	250	207
N-300	300	248

**Table 3:** Experimental and theoretical density values of HDPE composites with different fillers, along with the corresponding error percentages.

Sample Code	Composition (wt%)	Density (g/cm^3^) (Experimental)	Density (g/cm^3^) (Calculated)	Error (%)
H-1	Pure HDPE	0.957	0.950	0.69
HWC-2	HDPE +WC	1.102	1.106	0.35
HMoC-3	HDPE + MoC	1.087	1.096	0.85
HW-4	HDPE + W	1.091	1.108	1.49
HMo-5	HDPE +Mo	1.084	1.099	1.40

Note: The experimental densities were measured with an uncertainty of ±0.002 g/cm^3^, based on the accuracy and repeatability of the measurement setup.

**Table 4:** Mechanical properties of pure HDPE and its composite samples.

Sample	Tensile Strength (MPa)	Elongation at Break (%)	Young’s Modulus (MPa)
H-1	38.8 ± 4.9	593 ± 68	1838 ± 55
HWC-2	35.6 ± 1.6	5.54 ± 0.2	1889 ± 36
HMoC-3	36.1 ± 0.5	5.89 ± 0.9	1917 ± 67
HW-4	34.7 ± 2.2	6.48 ± 1.1	2000 ± 80
HMo-5	32.3 ± 2.0	5.97 ± 0.7	2150 ± 92

**Table 5:** Onset degradation temperatures and residual weights at 700 °C for HDPE composites containing 15 wt% of W, WC, Mo, and MoC fillers.

Sample	Onset Temperature (°C)	Residual Weight at 700 °C (%)
H-1	503	4.44
HWC-2	504	19.99
HMoC-3	496	18.30
HW-4	450	18.44
HMo-5	449	19.13

**Table 6:** Experimental and theoretical mass attenuation coefficients (µₘ) of HDPE composites containing 15 wt% of W, WC, Mo, and MoC across different photon energies.

	**H-1**			**HWC-2**			**HMoC-3**		
**Energy (keV)**	**Measured**	**Calculated (XCOM)**	**Error (%)**	**Measured**	**Calculated (XCOM)**	**Error (%)**	**Measured**	**Calculated (XCOM)**	**Error (%)**
47.9	0.21	0.21	0.81	0.93	1.12	16.62	1.05	1.24	15.28
65.2	0.20	0.19	4.69	0.65	0.59	11.25	0.58	0.62	7.07
83.3	0.17	0.18	5.79	0.68	1.15	22.15	0.36	0.39	6.68
100	0.15	0.17	10.98	0.57	0.77	16.21	0.26	0.29	12.53
118	0.17	0.16	0.61	0.51	0.55	7.66	0.25	0.24	3.17
165	0.14	0.15	3.18	0.30	0.30	0.15	0.17	0.18	5.42
207	0.14	0.14	0.33	0.22	0.22	2.53	0.14	0.15	4.62
248	0.13	0.13	2.15	0.16	0.18	11.34	0.13	0.14	2.48
	**HW-4**				**HMo-5**				
**Energy (keV)**	**Measured**	**Calculated (XCOM)**	**Error (%)**		**Measured**		**Calculated (XCOM)**	**Error (%)**	
47.9	1.20	1.18	2.20		1.19		1.37	13.43	
65.2	0.71	0.61	15.93		0.75		0.67	10.50	
83.3	1.02	1.21	15.78		0.42		0.42	1.55	
100	0.66	0.81	18.62		0.29		0.31	7.76	
118	0.50	0.58	14.01		0.23		0.25	6.40	
165	0.32	0.31	2.33		0.16		0.18	8.68	
207	0.19	0.23	15.83		0.15		0.15	1.59	
248	0.16	0.18	13.84		0.13		0.14	3.15	

**Table 7:** Measured radiation protection efficiency (RPE) values (%) at different incident photon energies for the studied samples.

Energy (keV)	H-1	HWC-2	HMoC-3	HW-4	HMo-5
47.9	45.00	94.23	96.46	98.05	97.32
65.2	43.69	86.48	84.03	90.24	91.07
83.3	38.39	87.68	68.65	96.43	74.70
100	35.34	82.76	55.94	86.36	60.47
118	37.59	78.90	54.35	75.76	52.96
165	33.71	60.44	40.92	56.86	41.03
207	32.72	48.24	36.61	46.37	37.28
248	30.47	38.57	34.39	40.29	34.92

**Table 8:** Buildup factors (B) for HDPE polymer composite samples.

Energy (keV)	H-1	HWC-2	HMoC-3	HW-4	HMo-5
47.9	0.67	0.15	0.11	0.07	- *
65.2	0.68	0.26	0.29	0.21	0.20
83.3	0.72	0.25	0.46	0.11	0.40
100	0.75	0.31	0.58	0.26	0.54
118	0.73	0.35	0.59	0.39	0.60
165	0.76	0.54	0.70	0.57	0.70
207	0.77	0.64	0.74	0.66	0.72
248	0.78	0.72	0.76	0.71	0.75

* A reliable B value for sample HMo-5 at 47.9 keV could not be obtained because the transmitted intensity was below the detection limit of the setup, even after repeated measurements.

**Table 9:** Effective atomic number (Z_eff_) of HDPE polymer composite samples.

Energy (keV)	H-1	HWC-2	HMoC-3	HW-4	HMo-5
47.9	2.818	65.886	44.755	66.963	38.469
65.2	2.752	61.090	39.898	62.631	36.077
83.3	2.722	56.016	35.415	57.954	33.401
100	2.695	64.468	9.366	65.631	27.520
118	2.688	61.804	9.047	63.227	24.732
165	2.678	53.880	8.539	55.917	18.871
207	2.674	46.259	8.282	48.654	15.277
248	2.672	39.928	8.151	42.438	13.164

## Data Availability

The original contributions presented in this study are included in the article/Supplementary Material. Further inquiries can be directed to the corresponding author.

## Supplementary Materials

The following supporting information can be downloaded at: https://www.mdpi.com/article/10.3390/polym18070893/s1, Figure S1. Representative stress–strain curves of HDPE containing 0 wt% (H1), and 15 wt% of WC (HWC-2), MoC (HMoC-3), W (HW-4), and Mo (HMo-5) particles. Figure S2. SEM-EDS images of pure HDPE (H-1). Figure S3. SEM-EDS images of HDPE composite containing a 15 wt% loading of WC (HWC-2). Figure S4. SEM-EDS images of HDPE composite containing a 15 wt% loading of MoC (HMoC-3). Figure S5. SEM-EDS images of HDPE composite containing a 15 wt% loading of W (HW-4). Figure S6. SEM-EDS images of HDPE composite containing a 15 wt% loading of Mo (HMo-5).
