# Supplementary material for: Mechanical, Thermal and X-Ray Shielding Properties of Lead-Free Composites of HDPE Filled with Metal-Based Powders
Source: Polymers (Basel). 2026 Apr 6;18(7):893. doi: 10.3390/polym18070893 (PMC13074431; doi:10.3390/polym18070893)
Supplement: Supplementary file 1 [file polymers-18-00893-s001.zip › polymers-4225347-supplementary.pdf]

# Mechanical, Thermal and X-ray Shielding Properties of Lead-Free Composites of HDPE Filled with Metal-Based Powders

**Sitah Alanazi[1], Shahad Alshadokhi[1], Eid Alosime[2], Mansour Almurayshid[2], Mohammed Alsuhybani[2 *], Mohammed Marashdeh[1]**

[1] Department of Physics, College of Sciences, Imam Mohammad Ibn Saud Islamic University (IMSIU), P.O. Box 90950, 11623, Riyadh, Saudi Arabia
[2] King Abdulaziz City for Science and Technology (KACST), Riyadh 11442, Saudi Arabia
* Correspondence: Dr Mohammed Alsuhybani; sohybani@kacst.gov.sa

Representative stress–strain curves have been provided in figure S1 to support and validate the reported tensile properties.

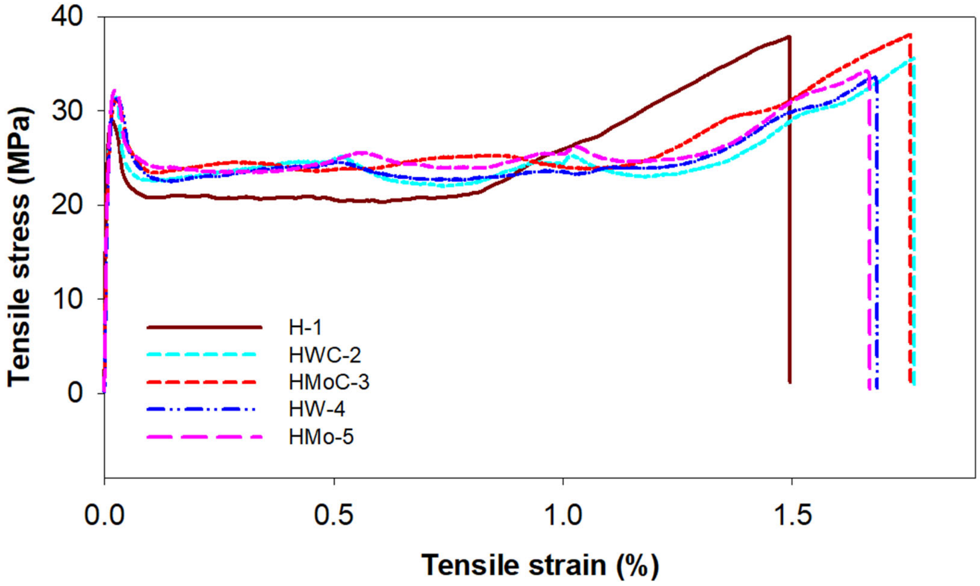


**Figure S1.** Representative stress-strain curves of HDPE containing 0 wt% (H1), and 15 wt% of WC (HWC-2), MoC (HMoC-3), W (HW-4), and Mo (HMo-5) particles.

SEM–EDS analysis was conducted to further confirm the presence, number, and spatial distribution of W, Mo, WC, and MoC atoms in the HDPE composites. Figures S2–S6 show the SEM–EDS results for neat HDPE (0 wt%) and HDPE reinforced with 15 wt% of WC, MoC, W, and Mo particles.

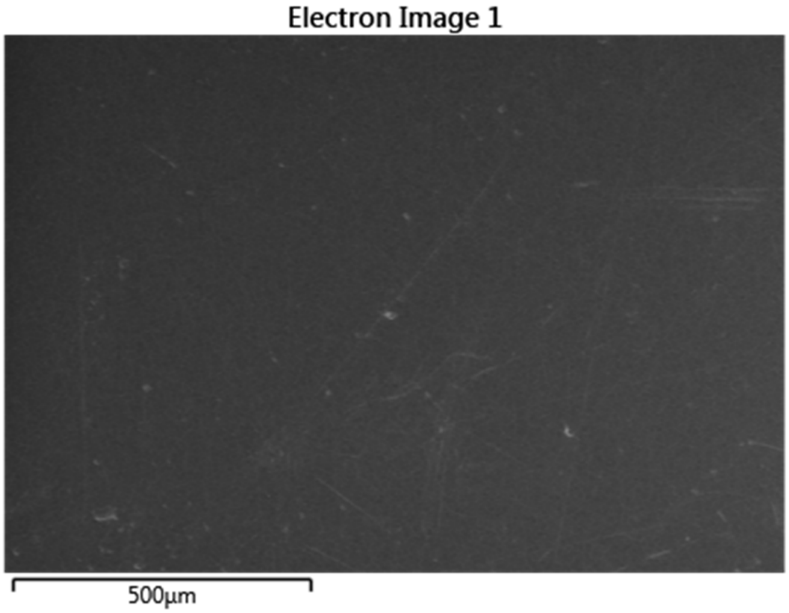


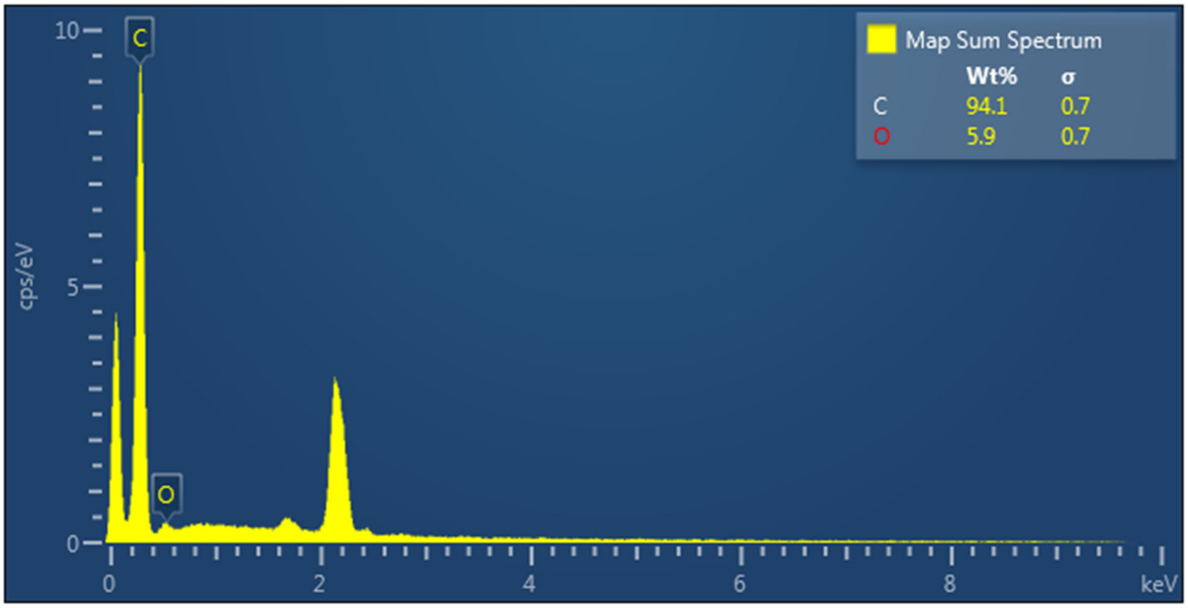


| Element | Wt% | Wt% Sigma |
|---|---|---|
| C | 94.14 | 0.68 |
| O | 5.86 | 0.68 |
| Total: | 100.00 | |

**Figure S2**. SEM-EDS images of pure HDPE (H-1).

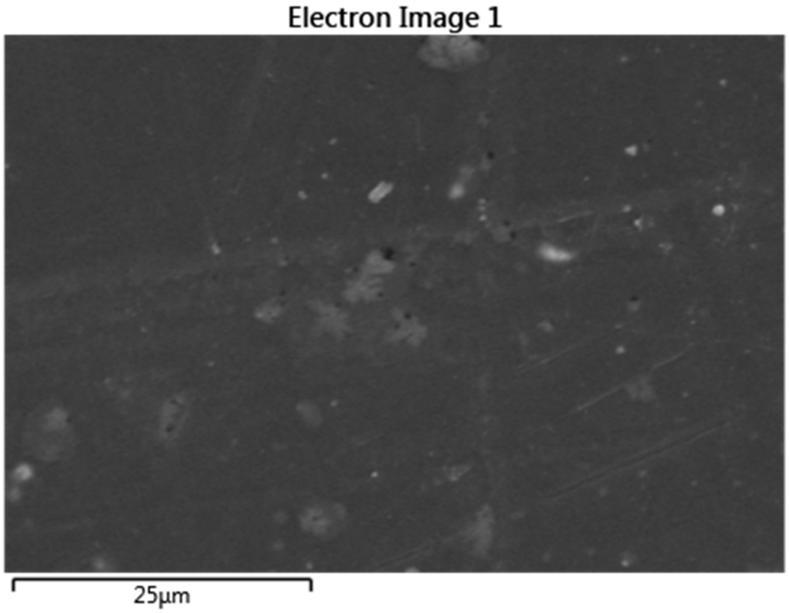


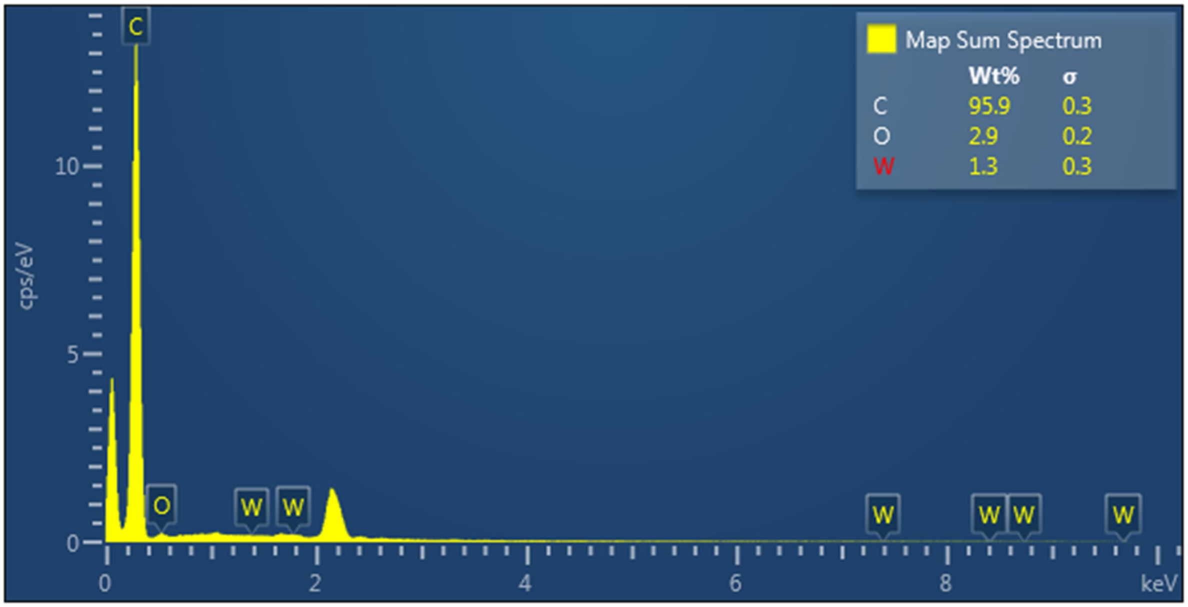


| Element | Wt% | Wt% Sigma |
|---|---|---|
| C | 95.87 | 0.34 |
| O | 2.87 | 0.22 |
| W | 1.26 | 0.27 |
| Total: | 100.00 | |

**Figure S3**. SEM-EDS images of *H*DPE composite containing a *15* wt% loading of WC (HWC-2).

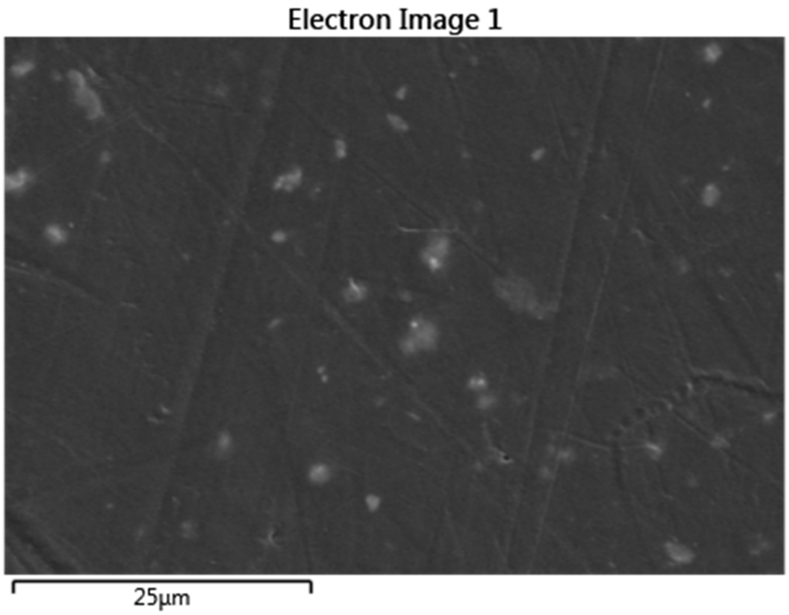


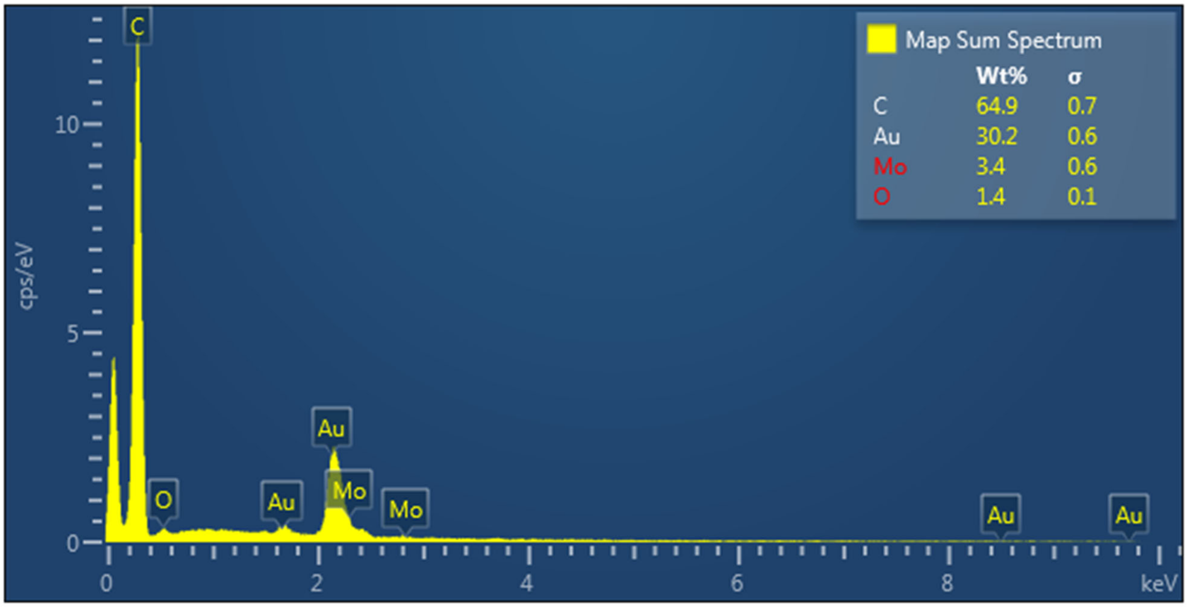


| Element | Wt% | Wt% Sigma |
|---|---|---|
| C | 64.92 | 0.66 |
| O | 1.43 | 0.15 |
| Mo | 3.43 | 0.58 |
| Au | 30.22 | 0.58 |
| Total: | 100.00 | |

**Figure S4**. SEM-EDS images of *H*DPE composite containing a *15* wt% loading of MoC (HMoC-3).

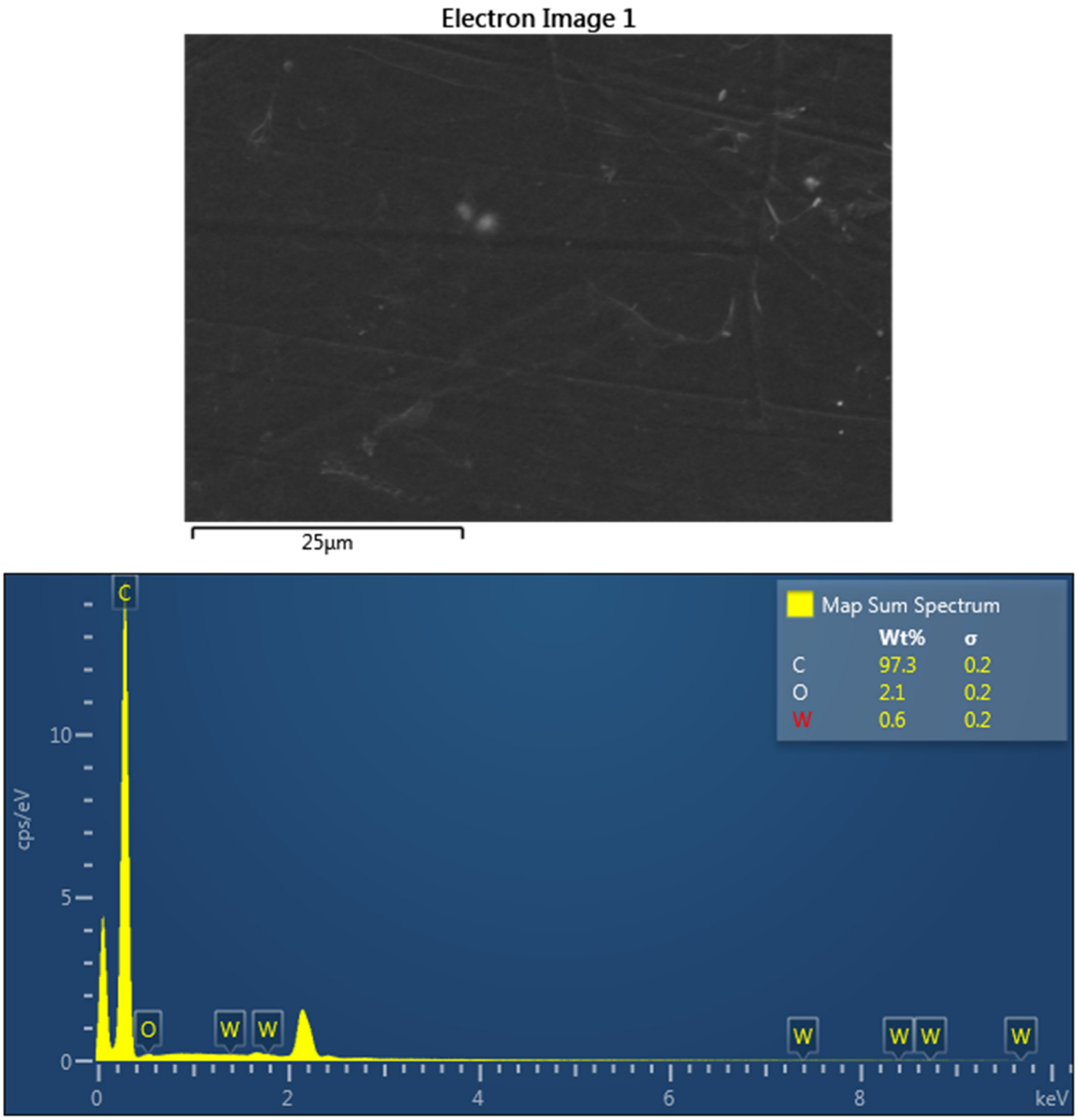


| Element | Wt% | Wt% Sigma |
|---|---|---|
| C | 97.30 | 0.24 |
| O | 2.05 | 0.16 |
| W | 0.65 | 0.19 |
| Total: | 100.00 | |

**Figure S5**. SEM-EDS images of HDPE composite containing a 15 wt% loading of *W (HW-4)*.

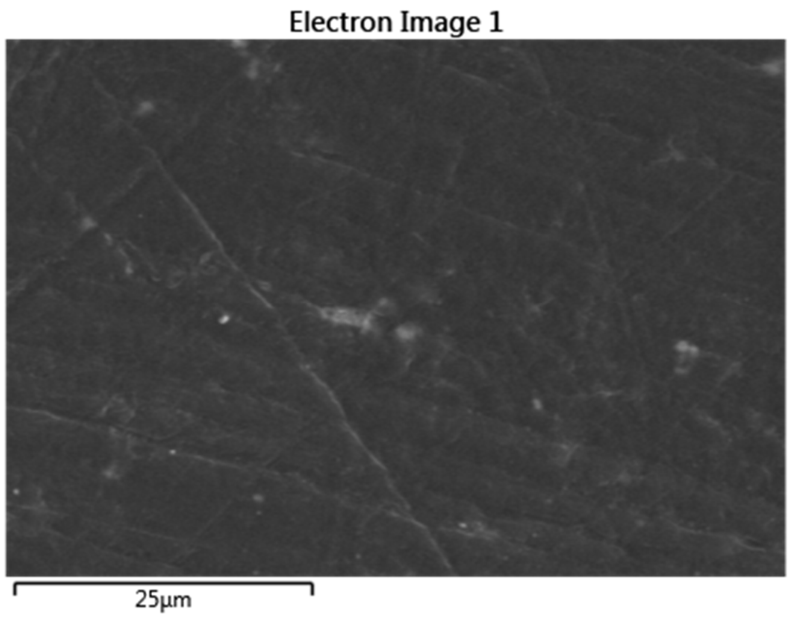


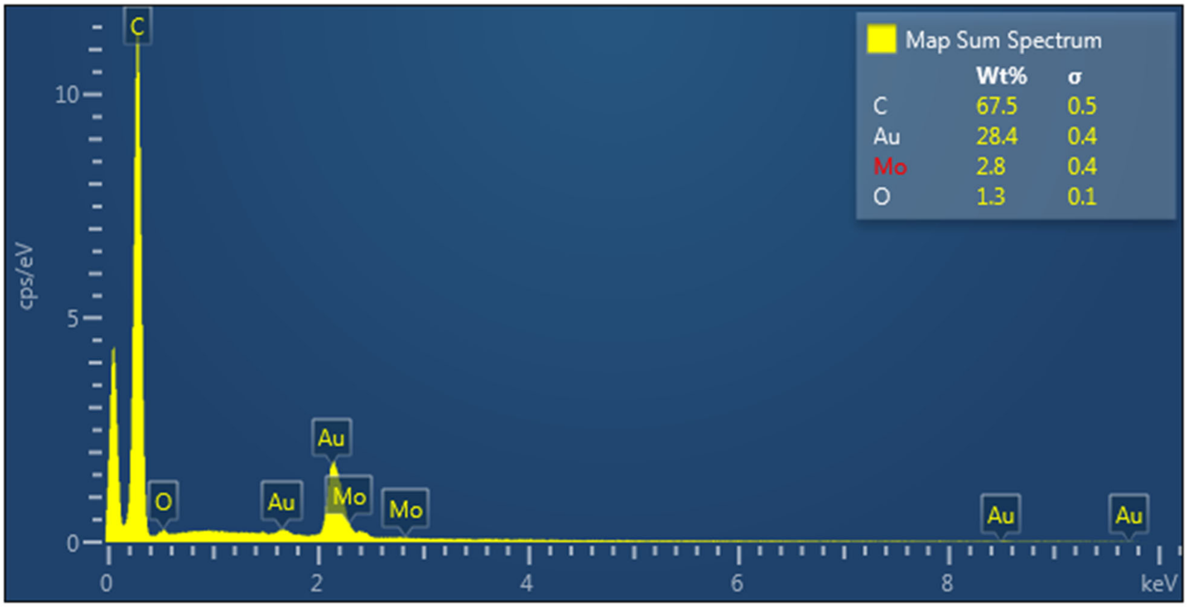


| Element | Wt% | Wt% Sigma |
|---|---|---|
| C | 67.49 | 0.50 |
| O | 1.33 | 0.11 |
| Mo | 2.76 | 0.42 |
| Au | 28.42 | 0.43 |
| Total: | 100.00 | |

**Figure S6**. SEM-EDS images of *H*DPE composite containing a *15* wt% loading of Mo (HMo-5).
